# Construction of a camelid VHH yeast two-hybrid library and the selection of VHH against haemagglutinin-neuraminidase protein of the Newcastle disease virus

**DOI:** 10.1186/s12917-016-0664-1

**Published:** 2016-02-26

**Authors:** Xiaolong Gao, Xiangyun Hu, Lina Tong, Dandan Liu, Xudong Chang, Haixin Wang, Ruyi Dang, Xinglong Wang, Sa Xiao, Enqi Du, Zengqi Yang

**Affiliations:** College of Veterinary Medicine, Northwest A & F University, Yangling, Shaanxi Province 712100 P. R. China

**Keywords:** Newcastle disease virus, VHH, HN protein, Yeast two-hybrid

## Abstract

**Background:**

Newcastle disease (ND), which is caused by the Newcastle disease virus (NDV), is one of the most important avian diseases in poultry. Since its discovery in 1926, ND has caused great economic losses to the world poultry industry and remains a threat to chickens and wild birds. Although a stringent vaccination policy is widely adopted to control ND, ND outbreaks still occur, and virulent NDV is sporadically isolated from chickens and wild birds. To study the pathogenesis of ND and provide tools to prevent its prevalence, novel antibody fragments should be developed. The variable domains of the heavy chain of the heavy-chain antibodies (VHH) are the smallest naturally occurring antibodies derived from camelid heavy-chain antibodies. The comparatively small size, high affinity, high solubility, low immunogenicity and ability to bind epitopes inaccessible to conventional antibodies of VHH make them ideal candidates for a considerable number of therapeutic and biotechnological applications. However, an anti-NDV VHH has not been reported to date.

**Results:**

In this study, a VHH yeast two-hybrid library was constructed from NDV vaccine immunized *C. bactrianus,* and seven VHH fragments to the haemagglutinin-neuraminidase (HN) protein of NDV were successfully screened and characterized for the first time. These selected VHH clones were all expressed as soluble protein in *E. coli*. ELISA, dot blot, immunocytochemistry and pull down results showed that the screened VHHs could interact with NDV virion, among which five had neutralizing activity. In addition, the seven VHHs could inhibit the haemagglutination activity of different NDV strains.

**Conclusions:**

We constructed an NDV-immunized VHH yeast two-hybrid library and screened and characterized seven VHHs targeting NDV HN protein for the first time. The seven VHHs may have great potential for NDV diagnosis, pathogenesis and therapeutics.

**Electronic supplementary material:**

The online version of this article (doi:10.1186/s12917-016-0664-1) contains supplementary material, which is available to authorized users.

## Background

Newcastle disease (ND), which is caused by the Newcastle disease virus (NDV), is one of the most serious avian diseases. NDV belongs to the family *Paramyxoviridae* and genus *Avulavirus.* NDV can infect a wide range of domestic and wild bird species and cause great economic losses to the poultry industry [1, 2]. It is an enveloped, single-stranded, non-segmented, negative-sense RNA virus with a genome length of approximately 15 kb nucleotides that contain six genes encoding for six structural proteins and two additional proteins [3]. Haemagglutinin-neuraminidase (HN) protein is one of the major glycoproteins. It forms spike-like structures on the outer surface of the virion and mediates the attachment of the virus to the sialic acid-containing receptors [4]. The HN protein is also an important target of host immune responses and a major protective antigen. Monoclonal antibodies of the HN protein were found to neutralize NDV infectivity [5]. Therefore, the HN protein is considered the most predominant antigen in the control of NDV.

In the past decades, ND epidemics were effectively controlled because of widespread vaccination. However, recent ND outbreaks in vaccinated flocks still caused damage to the poultry industry, and virulent NDV is continually isolated from vaccinated chickens [6–9]. A considerable number of studies indicated that current vaccines and therapeutic antibody-like biological agents could not completely stop the transmission of virulent NDVs [10–12]. Therefore, the development of novel methods for ND control is necessary.

The variable domains of camelid heavy-chain antibodies (VHH) are the smallest naturally occurring functional antibody fragments that maintain the antigen-binding capacity [13, 14]. Their comparatively small size, monomeric behavior, high stability, high solubility, powerful penetrability, low immunogenicity, and ability to bind epitopes inaccessible to conventional antibodies make VHHs ideal candidates for many therapeutic and biotechnological applications [15]. Therefore, the screening and characterization of VHH against NDV have great importance in ND control, discovery of potential epitopes, and antigenic variation research.

In this study, a VHH yeast two-hybrid library was successfully constructed from inactivated NDV vaccine-immunized *C. bactrianus*, and seven HN-targeted VHHs were obtained after screening. Deduced amino acid sequence alignment results indicated that the residues at position 37, 44, 45 and 47 of the amino acids sequence of the seven VHHs were also characteristic of a previously reported VHH, except VHH7. Using prokaryotic expression vector pHSIE, seven VHHs were expressed as a soluble recombinant protein fused with 6 × His-SUMO tag in *E. coli*. ELISA, dot blot and immnunocytochemistry results showed that the selected VHHs could specifically interact with the NDV. Haemagglutination inhibition assay (HI) showed that the seven selected VHHs could inhibit the haemagglutinin activity (HA) of NDV. Furthermore, five VHHs showed neutralizing activity against virulent NDV. The selected HN-targeted VHHs have potential for developing of NDV therapeutic and diagnostic reagents.

## Methods

### Viruses

La Sota and F48E9 strains of the NDV were used in this study. The La Sota is a widely used genotype II lentogenic vaccine strain. While the F48E9 is a standard genotype IX velogenic strain in China. These two strains were propagated in the allantoic cavity of 9–11 day-old specific pathogen-free embryonated chicken eggs. Allantoic fluid was harvested from embryonated chicken eggs and stored at −70 °C until use.

### Cells, yeast strains, and plasmids

DF-1 cells and HEK 293 T cells were maintained in Dulbecco’s modified Eagle’s medium (DMEM) supplemented with 10 % fetal bovine serum (FBS), 100 U/mL penicillin, and 0.1 mg/mL streptomycin. Plasmid pHSIE was kindly provided by Yi Zheng of the Shenzhen Key Lab of Gene and Antibody Therapy, Tsinghua University. Y2HGold yeast strain, Y187 yeast strain, pGADT7-Rec (cloning vector), pGBKT7 (cloning vector), pGADT7-T (control vector), pGBKT7-Lam (control vector), and pGBKT7-53 (control vector) were purchased from Clontech (Japan).

### Animal and vaccination

A six-month-old female *C. bactrianus* was immunized with a combination of inactivated NDV (La Sota) and subtype H9 avian influenza (Strain F) vaccine (Qingdao Yebio Bioengineering Co., Ltd, China) five times at two-weeks intervals. The administrated dose was based on the weight ratio between chicken and *C. bactrianus.* After vaccination, the humoral immune response was monitored by HI assay in V-bottom microtiter plates as previously described [16]. The animal with a strong response was bled 20 day after the last immunization.

### RNA isolation and VHH amplification

Approximately 70 mL immunized animal blood was collected 20 day after the last immunization. Lymphocytes were isolated with Ficoll-Paque PLUS and stored at −70 °C until use. Total RNA was extracted from approximately 10^7^ lymphocytes using the RNeasy Plus Mini Kit (Qiagen, Germany), and the first-strand cDNA was synthesized using the HiScript 1st Strand cDNA Synthesis Kit (Vazyme, China) with Oligo-dT primers. The first round of polymerase chain reaction (PCR) was performed with synthesized cDNA as a template using the primers V-F and V-R (Table 1) to amplify a 900 bp fragment encoding VH-CH1-CH2 and a 600 bp fragment encoding VHH-CH2. The 400 bp fragment of VHH was amplified through a second round of PCR using the gel-purified 600 bp fragment from the first round of PCR as a template with primers VHH-F and VHH-R (Table 1). The 400 bp VHH fragment was excised from the gel and purified using a gel extraction kit (OMEGA, USA).

**Table 1:** Primers used in this study

Primer name	Sequence^a^ (5’-3’)
V-F	GTCCTGGCTGCTCTTCTACAAGG
V-R	GGTACGTGCTGTTGAACTGTTCC
VHH-F	***TTCCACCCAAGCAGTGGTATCAACGCAGAGTGG***GAGTCTGGRGGAGG
VHH-R	***GTATCGATGCCCACCCTCTAGAGGCCGAGGCGGCCGAC***ATGGAGACGGTGACCWGGGT
T7	TAATACGACTCACTATAGGG
3’AD	AGATGGTGCACGATGCACAG
HN-F	CGGGATCCGTGGGGCTAGCACACCTAGCGAT
HN-R	GCGTCGACCTAGCCAGACCTGGCTTCTC
VHH-EF	CGGGATCCCAGGTGCAGCTGGTGGAGTCTGGRGGAGG
VHH-ER	GCGTCGACTTAGCTGGAGACGGTGACCWGGGT

^a^Letters in bold italics is the homologous arms

Underlined nucleotides in primers representing the restriction enzyme sites

### Yeast two-hybrid library construction and quality evaluation

Y187 yeast competent cells were prepared using the Yeastmaker Yeast Transformation System 2 kit according to the user manual. About 20 μL of VHH fragments (4–5 μg) and 3 μg of linearized pGADT7-Rec were co-transformed into Y187 yeast cells to construct the VHH Y2H library according to the Make Your Own “Mate & Plate™” Library System User Manual (Clontech, Japan). To determine the complexity of the library, 100 μL of 1/10, 1/100 and 1/1000 dilutions of transformed cells was spread on SD/–Leu (synthetically defined medium lacking leucine) 100 mm agar plates. After incubation at 30 °C for 3 −4 days, numbers of independent colonies that appeared on the dilution plates were counted to calculate library capacity. To determine the titer of the constructed VHH yeast two-hybrid library, 100 μL of library aliquot was taken out and serially diluted to 1/100, 1/1000, 1/10000, and 1/100000. The library titer was calculated according to the colonies appearing.

Simultaneously, 47 random clones were picked for insertion diversity identification by PCR using the universal primers 3’AD and T7 (Table 1). Ten positive clones produced a PCR product of approximately 400 bp were chosen randomly for gel-purification and sequencing. The sequence results were analyzed using the NCBI BLASTP program to verify the signature of the VHH insertions.

### Yeast two-hybrid screen

To obtain HN protein targeting VHH antibodies from yeast two-hybrid library, the bait protein with truncate coding sequences of HN (49–577 aa) from La Sota strain was cloned into the *BamH* I and *Sal* I sites of pGBKT7 and expressed as a fusion to the yeast GAL4 DNA-BD. Before screening, the bait protein was tested against the autoactivity and toxicity of HN. Then, a concentrated Y2HGold (pBD-HN) culture with 1 mL of Y187 (pAD-VHHs) yeast two-hybrid library aliquot was mixed for mating according to the Matchmaker™ Gold Yeast Two-Hybrid protocol (Clontech, Japan). Seventy 150 mm DDO/X/A (double dropout medium lacking tryptophan and leucine and supplemented with X-α-Gal and Aureobasidin A) plates were used to screen the clones after mating for 3−5 days. All blue colonies that appeared on DDO/X/A plates were then patched out and allowed to grow on QDO/X/A (quadruple dropout medium lacking adenine, histidine, tryptophan and leucine and supplemented with X-α-Gal and Aureobasidin A) plates. The blue colonies were screened twice on QDO/X/A plates to rescue the additional library plasmids and eliminate the false positives. The bait plasmid (pBD-HN) and rescued prey plasmid (pAD-VHH)s were co-transformed into Y2HGold yeast strain to confirm the interactions in yeast cells. The bait plasmid (pGBKT7-53 or pGBKT7-Lam) was co-transformed into Y2HGold with the prey plasmid (pGADT7-T) to serve as positive and negative controls, respectively. The rescued genuine positive AD/library VHH inserts were further sequenced and aligned using the NCBI BLASTP program.

### Expression and purification of VHH

The screened VHH fragments were amplified with the primers VHH-EF and VHH-ER (Table 1) and cloned into plasmid pHSIE using the *Bam*H I and *Sa*l I restriction enzyme sites for fusion with the 6 × His-SUMO tag at the N-terminus. After sequencing, the recombinant expression plasmids were transformed into *E. coli* Rosetta (Novagen) and incubated at 37 °C overnight. Subsequently, 1 mL of an overnight culture of cell was inoculated into 100 mL LB ampicillin broth and further incubated at 37 °C for 3–4 h (OD_600_ reached 0.4–0.6). The culture was induced by 0.5 mM isopropyl-D-thiogalactopyranoside (IPTG) at 30 °C for 5 h. The cell pellets were collected to check the expression of VHH antibodies by 12 % sodium dodecyl sulfate polyacrylamide gel electrophoresis (SDS-PAGE). The VHH antibodies were further purified using Talon Metal Affinity Resins (Clontech) according to the user manual and eluted with phosphate-buffered saline (PBS) containing 250 mM imidazole.

### HI assay

The specific interaction between purified VHH and HN was tested using HI assay as previously described [16]. Briefly, 25 μL of PBS containing 4 HA units of antigen was incubated in a V-bottom 96-well plate with 25 μL of twofold serially diluted VHHs at room temperature for 40 min. Subsequently, 50 μL of 1 % suspension of chicken erythrocytes was added. The mixture was incubated at room temperature for 30 min. The purified bacterial lysate of bacteria transformed with an empty pHSIE vector or an anti-NDV positive serum were used as negative and positive controls, respectively. Agglutination of erythrocyte in the plate was scored. HI assay was performed using different strains. Titers were calculated as the highest reciprocal dilutions that completely inhibited haemagglutination. All tests were performed twice.

### ELISA

To verify the interaction between the selected VHH and NDV, the NDV La Sota strain was coated onto Maxisorp 96-well plates (Nunc) in Na_2_CO_3_ (0.05 M, pH 9.6) overnight at 4 °C with 100 μL/well. The purified 6 × His-SUMO tag and anti-NDV negative serum served as the negative control, and the anti-NDV positive serum (kept by our laboratory) served as the positive control. In the following, the plates were blocked with 5 % skimmed milk–PBS with 0.05 % Tween 20 (PBST) for 1 h at 37 °C. After rinsing for five times with PBST, 100 μL diluted VHH (200 μg/mL) was added into each well for 2 h further incubation at 37 °C in triplicate. Subsequently, the wells were rinsed five times with PBST, and 100 μL of horseradish peroxidase (HRP)-conjugated Anti His-Tag Mouse Monoclonal Antibody (CWBIO, Beijing) was added at a 1:3000 dilution for 1 h at 37 °C. whereas, the positive control wells were treated with HRP-conjugated goat anti-chicken antibody (Bioss, Beijing). After washing with PBST for five times, the reaction was developed by adding 100 μL tetramethylbenzidine (TMB) and stopped by adding 50 μL 2 N H_2_SO_4_ after 20 min of incubation at room temperature. The absorbance was read using a microplate reader at 450 nm (Bio-Rad).

### Dot blot analysis

Dot blots were performed according to western blot protocols, except that sucrose-gradient centrifugation purified NDV were spotted on a polyvinylidene fluoride membrane (0.45 μm, Millipore, USA) without preceding gel electrophoresis. The membrane was blocked with 5 % skimmed milk for 2 h at room temperature and then washed twice with TBST (Tris buffered saline containing 0.5 % Tween 20). The purified VHHs (200 μg/mL) were applied at 1:100 dilution. After 2 h incubation at 37 °C, the membrane was washed four times and incubated with HRP-conjugated anti His-Tag mAb (1:5000 dilution) for 1 h at 37 °C. After four further washes with TBST, the immunoreactive was visualized with cECL Plus Western blotting detection reagent (CWBIO). The anti-NDV positive serum and negative serum were used as positive and negative controls.

### Pull down

To determine the interaction between VHH and HN protein, HEK 293 T cells were seeded in a 6 wells plate. The next day, 293 T cells were transfected with pCAGGS-HN plasmid or pCAGGS empty plasmid using lipofectamine 2000 reagent (invitrogen). Cells were put on ice 36 h post transfection, washed once with PBS and subsequently lysised with 150 μL/well RIPA buffer for 10 min. The lysate was centrifugated for 5 min at 13000 rpm to remove cell debris. The supernatant was used as cell derived HN protein.

1000 μL of the 293 T-HN lysate was incubated with 80 μg VHH, 500 μL talon Metal affinity resin (Clontech) and 4 mL PBS for 2 h at 4 °C. Talon matrix was washed twice with 4 mL PBS. 300 μL sample buffer was added, boiled for 10 min and separated with SDS-PAGE. The gel was blotted and the membrane was immunolabeled with guinea pigs anti-HN polyclonal antibody (prepared by our lab), HRP goat anti-Guinea pig IgG (ABclonal) and HRP-conjugated anti His-Tag mAb (CWBIO).

### Neutralizing assay

Virus neutralization (VN) assay was conducted to confirm the neutralizing activities of the selected VHH. Briefly, 50 μL of the purified VHH (200 μg/mL) was twofold serially diluted and then incubated for 1 h at 37 °C with 100 TCID_50_ of the NDV F48E9 strain in DMEM. After incubation, the DF-1 monolayer of cells grown in 96-well tissue culture plates was infected with the virus-VHH mixture. After absorption at 37 °C for 1 h, the cell cultures were washed twice with PBS. Fresh DMEM with 2 % FBS was subsequently added. The cell cultures were further incubated for 36 h at 37 °C. Syncytium formation of the infected cells was observed in each well. Virus- and mock-infected cells were used as controls. Anti-NDV positive and negative serum were also included as positive and negative controls, respectively. Neutralizing activity was primarily confirmed by the absence of syncytium formation.

To further quantify the neutralization activity, supernatants of cell cultures were harvested at 36 h post inoculation, serially diluted, and assayed for virus titer using Reed-Muench method [17].

### Immunocytochemistry assay

To verify the specific intracellular binding activity between selected VHHs and NDV virus, LaSota-infected DF-1 cells and mock control were grown on 24-well dishes for the immunoperoxidase assay. After 48 h, the cells were fixed with 4 % paraformaldehyde on 24-well dishes and were permeabilized with PBS-0.5 % Triton X-100. The fixed cells were incubated with blocking solution (2 % BSA) followed by the VHH for 2 h at 37 °C. Subsequently, the treated cells were incubated with horseradish peroxidase (HRP)-Conjugated Anti His-Tag Mouse Monoclonal Antibody (CWBIO, Beijing) at a 1:4000 dilution for 1 h at 37 °C. The detection of the antigen was carried out using DAB Kits (CWBIO). At last, the nuclear of cells were stained by hematoxylin. The results were examined under an inverted light microscope.

### Statistical analysis

Statistically significant differences in HI assay and ELISA assay among VHHs were evaluated using Student’s *t* test with the Prism 5.0 (Graph Pad Software, Inc., San Diego, CA). The calculated P values of < 0.05 were considered statistically different.

### Ethics statement

*C. bactrianus* was grown in a farm of the Gobi Desert, northwest of China, with feed and water administrated *ad libitum.* The inactivated vaccine immunizations and jugular vein blood sampling were performed in strict accordance with the Animal Ethics Procedures and Guidelines of the People's Republic of China. The study was approved by the Animal Ethics Committee of Northwest A&F University, Shaanxi Province, China. In this study, the owner of the *C. bactrianus* gave us permission to experiment on the animal, which was returned to the owner after the completion of the experiment. We unequivocally state that this work did not involve any ethical issues.

## Results

### Anti-NDV specific HI antibody titer of camel serum

Approximately 5 mL blood of was aseptically collected from the immunized animal 14 day after second and fifth immunization to evaluate the humoral response. The blood was allowed to clot to extract the serum. Then, the serum was used for HI assay with 4 HA units of La Sota strain as the antigen, as described in the OIE manual [16]. The anti-NDV HI antibody titer was 10 log2 (Additional file 1: Table S1).

### VHH amplification and yeast two-hybrid library construction

As shown in Additional file 1: Figure S1A, a 900 bp fragment and a 600 bp fragment were successfully amplified with the primers V-F and V-R in the first round of PCR. Then, the 400 bp VHH fragment was amplified using the gel-purified 600 bp fragment from the first round of PCR as the template with the primers VHH-F and VHH-R through a nest PCR (Additional file 1: Figure S1B).

During the library construction, 4.4 μg gel-purified VHH fragment was co-transformed with 3 μg *Sma* I linearized pGADT7-Rec plasmid into Y187 competent cells for recombination. Three parameters were used to evaluate the quality of the yeast two-hybrid library: library capacity, library titer, and percentage of library clones that contain insertions. Results of the calculations were as follows: the library capacity was 1.25 × 10^7^(Additional file 1: Figure S3A), and the library titer was 3.45 × 10^8^ cfu/mL(Additional file 1: Figure S3B). A total of 45 clones in 47 randomly selected clones had 400 bp VHH bands after PCR identification with primers T7 and 3’AD (Additional file 1: Figure S2); thus, the percentage of library insertion rate was approximately 96 %. Ten PCR positive clones were chosen randomly for sequencing. The results showed that these clones were all unique sequences, indicating diversity of the library (Additional file 1: Figure S4). Thus, the quality of the VHH yeast two-hybrid library met the demand for screening.

### Bait construction

The truncate HN gene was amplified from the cDNA of the La Sota strain using the primers HN-F and HN-R. The amplified products were approximately 1600 bp (Additional file 1: Figure S5A). Subsequently, the amplified fragments were subcloned into vector pGBKT7 by *BamH* I and *Sal* I digestion and ligation (Additional file 1: Figure S5B). Clones were verified by sequencing. Finally, the pGBKT7-HN was transformed into Y2HGold yeast strain as a bait.

### Selection of HN-specific VHH antibody fragments

**Fig. 1:**
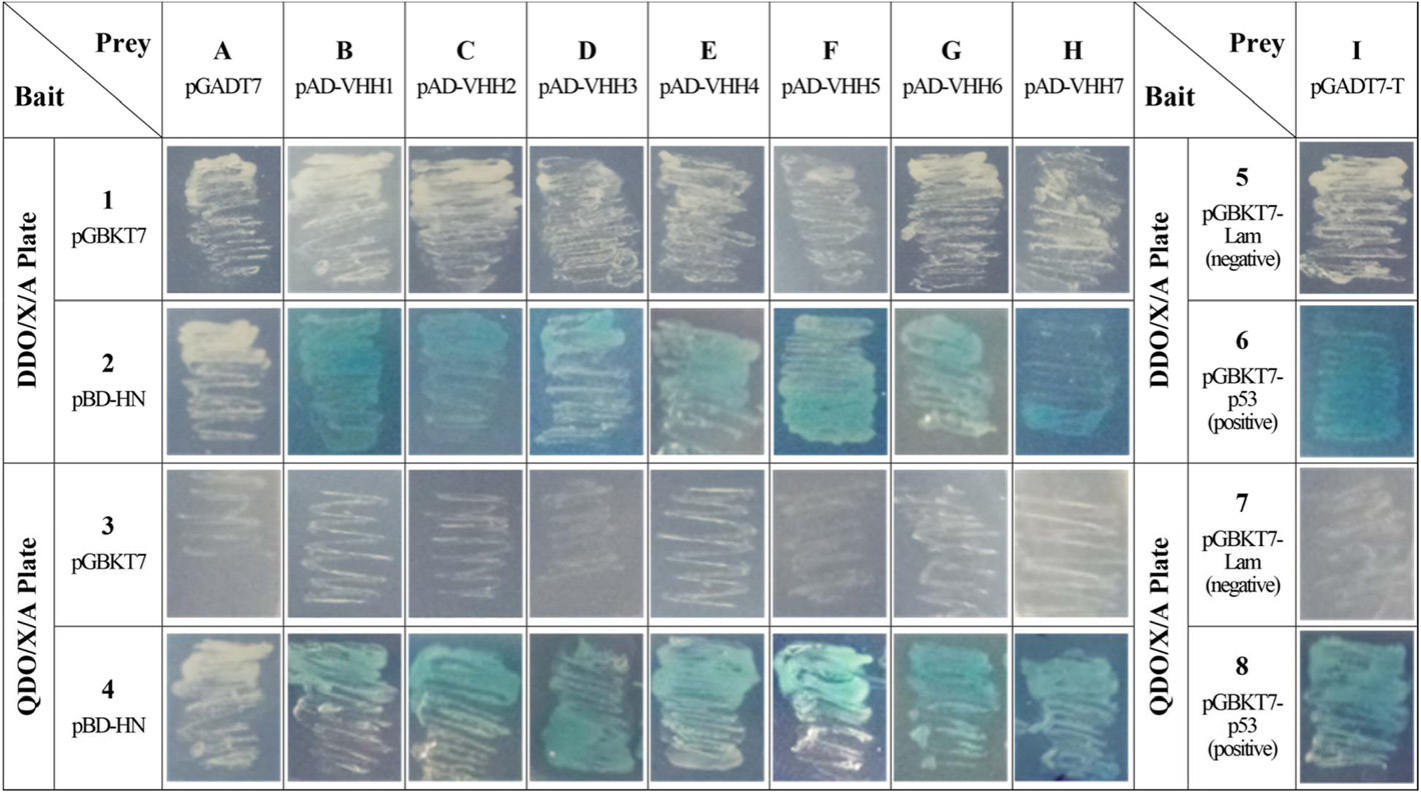
Interaction between HN and VHH in yeast. The white clones in lane A showed that the bait(pBD-HN) has no autoactivator activity in yeast. When pBD-HN and 7 positive pAD-VHH were cotransformed into Y2HGold yeast strain, all cotransformants turned blue on DDO/X/A and QDO/X/A plates after incubation. At the same time, the pGBKT7-p53 and pGADT7-T co-transformants was used as positive control, whereas pGBKT7-Lam and pGADT7-T co-transformants was used as negative control

**Fig. 2:**
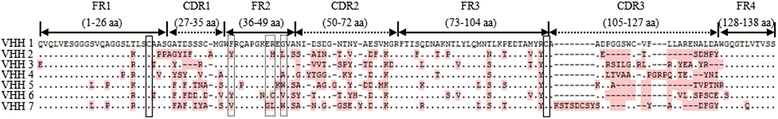
Deduced amino acid alignment of 7 selected anti-HN VHH. Deduced amino acid sequences were analyzed according to the Kabat numbering [54]. The dots indicate the same sequences compared with VHH1. Differences in the sequences are pinked, and the dash represent the missing sequences. Two hallmark Cys residues are labeled by the thick-line boxes. The four conservative hallmark residues of VHH in FR2 are labeled by the dotted line boxes

Before screening, no autoactivator activity of the BD-HN was confirmed in yeast (Lane A, Fig. 1). When zygotes formed after mating the Y2H Gold (pBD-HN) and the Y187 yeast library (pAD-VHHs), the mating products were spread on the DDO/X/A plates for incubation at 30 °C for 3–5 days. Hundreds of blue yeast colonies were selected. However, only seven clones were recovered after two rounds of screening on the QDO/X/A plates. To verify the positive interactions between VHHs and HN protein in yeast, the prey plasmid isolated from the seven clones were co-transformed with the bait plasmid (pBD-HN) or the pGBKT7 empty vector. The co-transformation results showed that all the seven clones rescued from the QDO/X/A plates were genuine positive clones (Lanes B–H, Fig. 1). The sequencing results showed that the seven positive clones were of different clone origins and that the insertions were homologous to the properties of camel VHH, except VHH7 (Fig. 2).

### Expression and purification of VHH

**Fig. 3:**
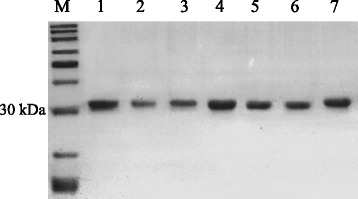
SDS-PAGE of purified VHH. All seven purified VHH were separated on 12 % SDS-PAGE gels. And the gel was stained with coomassie blue. Lane 1 to 7 represent VHH 1 to VHH 7, respectively. The molecular weight of the 6 × His-SUMO-VHH fusion proteins were about 33 kDa in size

Seven VHH fragments were cloned into pHSIE using the *BamH* I and *Sal* I restriction enzyme sites for expression and purification (Additional file 1: Figure S6). As 6 × His-SUMO tag was fused with the VHH fragments at the N-terminus, the recombinant VHHs were purified using Talon Metal Affinity Resin (Clontech). After electrophoresis on a 12 % SDS-PAGE, the 6 × His-SUMO-VHHs fusion proteins exhibited bands that were approximately 30 kDa (Lanes 1–7, Fig. 3). All VHH fragments were solubly expressed.

### HI titer of VHH

The HI assay is an important and convenient method widely used for antibody detection. As shown in Table 2, all selected VHHs shared an HI titer ≥ 4 log2 except VHH2, and the HI titer of the VHH was significantly lower than that of the positive serum. The HI titers, which were tested using different NDV strains as 4 HAU, were slightly different.

**Table 2:** HI titer of selected VHH

Strain	Origin	Genotype	HI titer (X Log2)								
			VHH1	VHH2	VHH3	VHH4	VHH5	VHH6	VHH7	Posivtive^a^	Negative^b^
F48E9	Chicken	IX	4	1	5	5	5	5	4	9	0
LaSota	Chicken	II	4	2	4	6	6	4	4	8	0
YuLin	Chicken	IX	4	1	5	5	5	5	5	8	0
Dove	Chicken	IX	4	1	4	4	4	5	4	6	0
W2	Chicken	VII	6	1	5	4	4	5	4	6	0

^a^Represent anti-NDV positive serum

^b^Represent sumo-tag

### ELISA

**Fig. 4:**
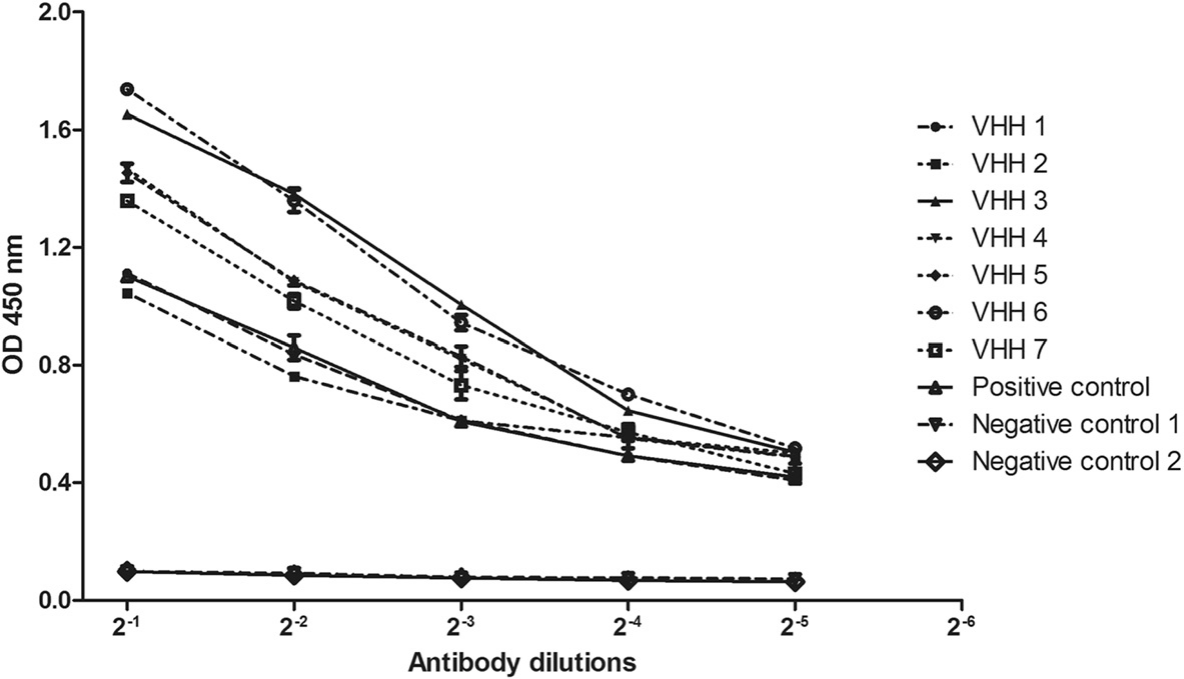
Detection of interaction between selected VHH and NDV virion by ELISA. Microplates were coated with NDV La Sota virion (TCID_50_ 10^-8.17^/0.1 mL, 1:20 dilution). The 7 selected VHH (200 μg/mL) were diluted at different concentrations, and anti-NDV positive serum (Positive control), negative serum (Negative control 1) and 6 × His SUMO tag (Negative control 2) were used as positive and negative control. The value of absorbance at 450 nm indicate the reactivity between VHH and NDV. And the data represents the average of 3 wells

Indirect ELISA was conducted to verify the specific interaction between the VHH and the NDV. For the plate coated with the NDV La Sota (TCID_50_ = 10^-8.17^/0.1 mL), the optimized coated concentration was 1:20. ELISA results showed that all the selected VHHs could react with the NDV virions (Fig. 4). The OD_450_ of the seven VHHs were significantly stronger than the negative controls (*P* < 0.05; Fig. 4). The reaction in the wells of the negative control was very weak (OD_450_ < 0.10). All tests were performed three times.

### Dot blot

**Fig. 5:**
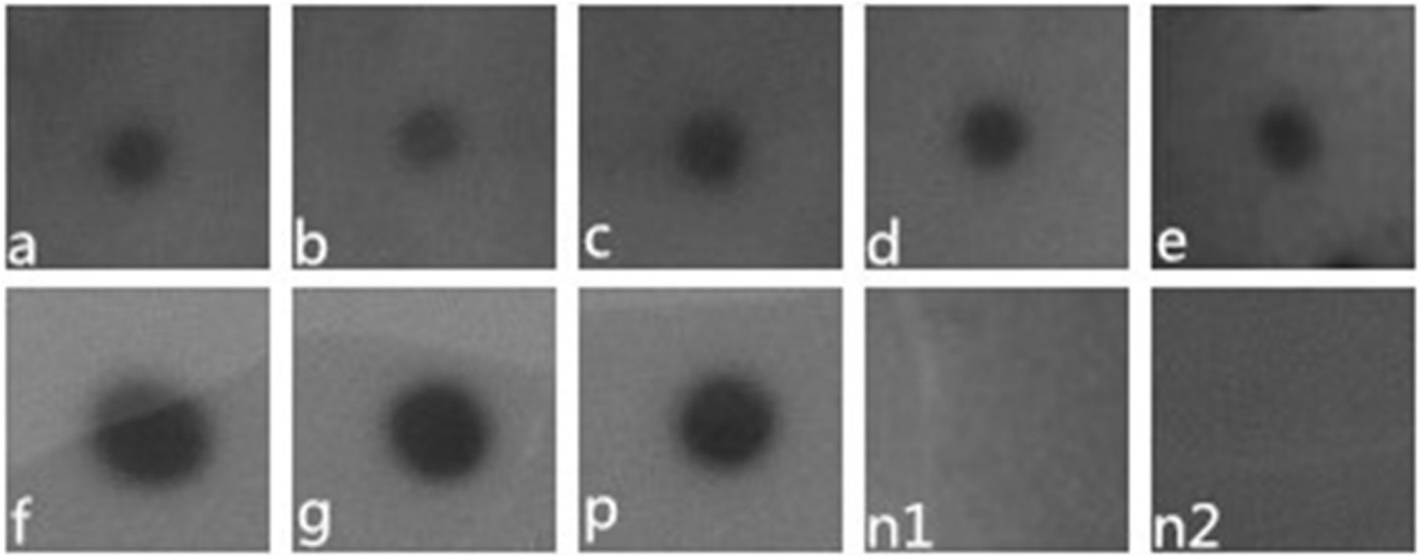
Detection of interaction between VHH and NDV virion by Dot blot. Purified NDV (a-p) or allantoic fluid (n1 and n2) were spotted on a PVDF membrane for blot. VHHs binding were visualized using HRP labeled anti-His Mabs While positive serum was visualized using HRP labeled anti-chicken antibody. a-g represent VHH1-VHH7; p represent positive serum; n1 represent VHHs (VHH 1 to VHH 7); n2 represent positive serum

The seven VHHs and positive serum could bind native NDV virion (Fig. 5, a-g and p). While the positive serum and VHHs could not bind allantoic fluids without NDV (Fig. 5, n1 and n2). This result suggested that the VHHs could recognize NDV.

Pull down

**Fig. 6:**
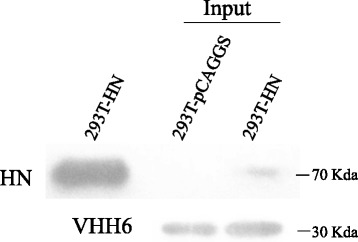
Validation of VHH against cell derived HN protein. Pull down of cell derived HN protein from pCAGGS-HN transfected 293 T cells lysate or pCAGGS empty vector transfected 293 T cells lysate using VHH6 with talon metal affinity resin. Western blot with anti-HN polyclonal antibodies and anti-his tag mAb

Using VHH6 as model, the interaction between VHH and HN protein was detected by His-tag Pull down assay. As shown in Fig. 6, VHH6 were able to pull down the HN protein from a HN transfected 293 T cell lysate and not from a pCAGGS empty vector transfected 293 T control cell lysate. The result indicated that these VHH6 could interact with HN protein.

### Neutralizing assay

**Fig. 7:**
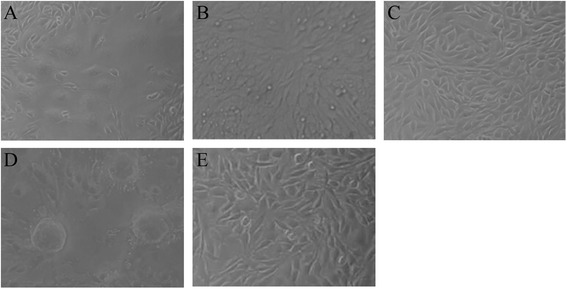
Syncytium formation of DF-1 cell treated with anti-HN VHH. Virus neutralizing assay was performed on DF-1 cells. Syncytium formation was observed under a microscope 36 h post inoculation. **a** 6 × His-SUMO tag treated. **b** VHH treated. **c** Anti-NDV positive serum treated. **d** Negative serum treated. **e** Mock

Neutralizing assay was performed to evaluate the neutralization ability of the VHHs against F48E9 infection. As shown in Fig. 7, the five VHHs (VHH3, VHH4, VHH5, VHH6, and VHH7) had neutralizing activity to inhibit the replication of F48E9 on the DF-1 cell as there was no syncytia appeared at 24 h post-inoculation, although the neutralizing titer was significantly lower than the positive control (VHH ≤ 2^2^,positive serum > 2^7^). However, some syncytia can be observed on cells at 36–72 h post-inoculation in experiment wells, while positive serum can effectively inhibit the infection of virus. The negative control showed no neutralizing activity.

**Fig. 8:**
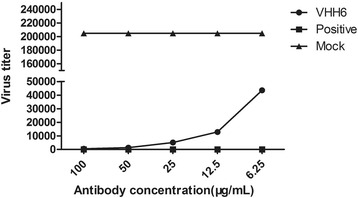
The determination of the effects of VHH on the virus production. Virus titer in the culture supernatants was measured at different VHH concentration by Reed-Muench method

VHH6 was picked out as an example to quantify neutralization activity. Thirty-six hour post-inoculation, supernatants were harvested and tested for virus titer. Results indicated that virus propagation was significantly inhibited by the VHH6 (Fig. 8).

### Detection of NDV-infected cells by immunocytochemistry

**Fig. 9:**
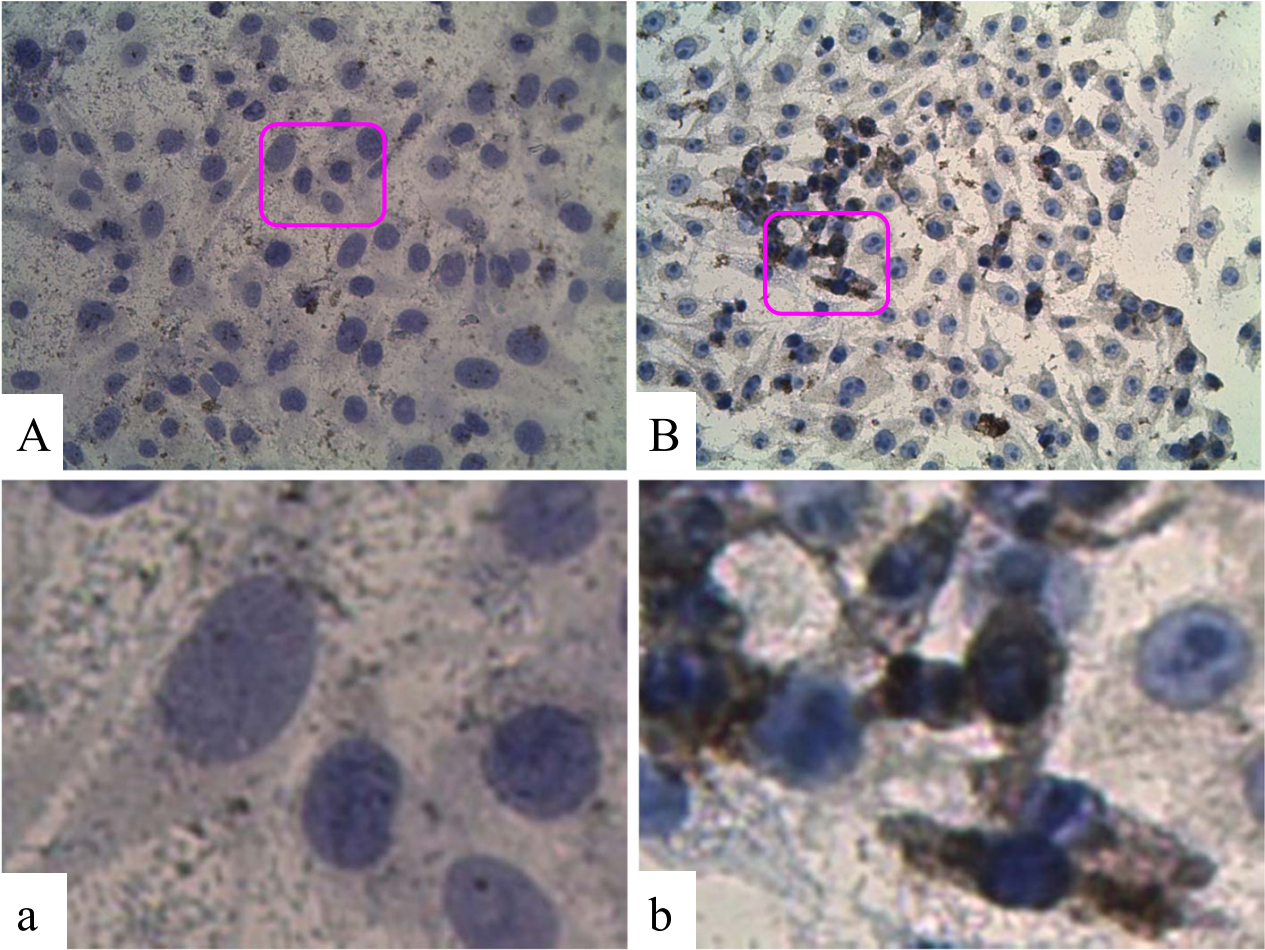
Immunocytochemistry analysis with VHH6. A and a, mock-infected DF-1 cells; B and b, LaSota infected DF-1 cells. All cells were examined under an inverted light microscope (40×). The expression of NDV HN glycoprotein is visualized as a brown color in the cytoplasm, and cell nuclei stained with hematoxylin are shown in blue in the DF-1 cells

Using VHH6 as model, the intracellular specificity and binding affinity of selected VHHs were further detected by immunocytochemistry. As shown in Fig. 9, the strong cytoplasm brownish staining was specifically appeared in NDV-infected DF-1 cells (0.01 MOI) group but not in the mock control. These results indicated the feasibility of detection intracellular NDV antigen by using selected VHHs.

## Discussion

The HN protein, which is an important multifunctional surface glycoprotein consists of a cytoplasmic domain, a transmembrane region, a stalk region and a globular head, possesses both HA and NA activities and all the antigenic sites [18]. HN protein binds to the sialic acid receptor mediating virus attachment to cells and promotes the fusion activity of the F protein, allowing the virus to penetrate the cell surface, and receptor-destroying activity to facilitate virus budding. The polyclonal or monoclonal antibody targeting HN protein has been reported to neutralize the infectivity of the NDV. Therefore, the HN protein is an ideal target for NDV treatment and many therapeutic reagents have been developed against HN protein, including hyper-immune sera, egg yolk antibody, and monoclonal antibodies [19–23]. However, anti-HN VHH have not been reported yet.

A phage display library is widely used to select scFv and VHH from a considerable number of interested targets, such as Influenza A virus M2 ion channel, chemokine receptor CXCR4, and murine ADP-ribosyltransferase C2 [24–26]. Acquiring pure antigen is the premise to panning a phage library. However, not all antigens are easy to prepare, and the process is usually time consuming and laborious. Yeast two-hybrid technology is a useful tool to study protein-protein interactions, including antibody-antigen interaction. A previous study reported the feasibility of using yeast two-hybrid to screen high affinity VHH against the targeted protein *in vivo*, which avoid the time-consuming antigen preparation [27]. In the present study, in order to avoid HN protein preparation, the truncate HN gene was cloned into bait plasmid for fusion expression with binding domain (BD) of Gal4. Then the autoactivity and toxicity of the bait protein were assessed, result showed that HN-BD had no autoactivity and toxicity. Whereas, the expression level of HN-BD in yeast Y2H Gold cells was relatively low (Additional file 1: Figure S7). The low expression level may be ascribed to the following reasons. On the one hand, HN protein contains 12 cysteines, the Y2H yeast strain might have problems in the production of proteins with multiple cysteine bridges. On the other hand, the host yeast cells, although a eukaryote, is distant from human, mamalian, and higher eukaryotic organisms. So, sometimes the conditions in yeast cells may not allow the proper folding or posttranslational modifications required for some proteins. Although in a low expression level, after screening the VHH yeast two-hybrid library with the truncate HN bait protein, seven VHHs were successfully obtained for the first time. HI assay, dot blot, ELISA, immunocytochemistry and pull down results indicated that the purified VHHs could react with the NDV virions or HN protein.

The VHH is generated from a V-D-JH gene rearrangement using a specific set of V gene segments, which exist in the germline of the camelid genome [28]. At least four distinct VHH subfamilies have been defined, but they all share several crucial amino acid substitutions in the framework 2 region, namely, Val37-Phe/Tyr, Gly44-Glu/Gln, Leu45-Arg, and Trp47-Gly/Phe/Leu (Kabat numbering), which increase the hydrophilicity of the VHH. These VHH hallmark residues compensate for the possible instability, hydrophobicity, and aggregation tendency of VHH because of the lack of VL domains [13, 29, 30]. The variable domains of antibody consist of four framework regions (FRs) and three complementary determining region (CDRs) [31, 32]. The amino acid sequences of the FR are generally constant and not easily changed. The amino acid sequences of the CDR are highly variable, and they determine the antibody specificity and diversity [33]. In this work, we screened seven VHH antibodies from *C. bactrianus* for the first time. The sequence alignment results showed that the four conservative hallmark residues of the VHH in FR2 were in accordance with previous reports [34], except for VHH7. By analyzing the alignment results we also found that the sequence differences of these VHHs were mainly localized in the CDRs as others reported [28, 30].

To avoid VH contamination in VHH library, usually two rounds PCRs are performed. The first is used to discriminate VH (VH-CH1-hinge-CH2) from VHH (VHH-hinge-CH2) based on amplicon sizes which differ by 300 bp in length. The second is used to amplified VHH from the short amplicons (VHH-hinge-CH2) with primers annealing at the codons of FR1 and FR4. Afterward VHH fragments are cloned for library construction and selection. In the present study, two successive PCRs were performed during IIama VHH library construction to select for VHH sequence rather than VH. After screening, seven positive clones were obtained. However, sequence alignment result showed that especially VHH7 did not contain the hallmark residues in FR2 that are typical of VHHs as described in previous reports [34]. The presence of variable single domains that seem to originated from VH have been repeatedly identified together with canonical VHHs in immune libraries [35, 36]. Monegal reported that single domains antibodies with VH hallmarks could be positively selected during panning of VHH libraries [37]. These unexpected VH sequences could result from PCR crossover between VHH and VH fragments or rearrangement of classical VH domains to IgG2 or IgG3 constant regions [38].

To investigate the reactivity of the selected VHHs with the NDV, ELISA, HI, dot blot and immunocytochemistry were performed. As shown in Fig. 4, all the selected VHHs could specifically interact with the NDV La Sota strain, and the reactivity were significantly stronger than negative controls (*P* < 0.05). The ELISA results suggested that all the selected VHHs could interact with NDV virions. HI assay is a convenient method widely used for the detection of interactivity between antibody and HN protein. As shown in Table 2, all VHHs could inhibit the HA activity of different strains, but they shared different HI titers against different strains. The different HI titers indicated that antigenic variations exists among different NDV strains, which is consistent with previously reported [39–41]. Whereas, the HI titers of VHHs were significantly lower than that of the positive serum. Previously, it has been demonstrated that HA activity was determined by multiple sites on HN protein [42–45]. Therefore, the lower HI titer of the VHH compared with the positive serum may be attributed to the fact that each selected VHH only recognized a limited number of epitopes associated with the HA activity. Moreover, the small size of the VHH may not be sufficient to form a steric hindrance that will completely block the active site involved in HA activity when binding on the HN. Multimerization or polyvalence of the VHH may be a promising method to enhance its function in the future.

Using monoclonal antibodies, seven antigenic sites have been identified in the HN glycoprotein and formed a linked continuum, in the order of sites 4-14-1-12-1-23-3 [5, 46]. And most of the HN epitopes were conformational epitopes, only one linear epitope was revealed (residues 345 to 353 aa) [5]. In the present study, we proved that all the selected VHHs could interact with NDV through dot blot, ELISA, and HI. At the same time, the VHH were also able to pull down HN protein from cell lysate. However, the reactivity were not detected using western blot (data not shown). These results suggested the VHHs might recognize conformational eipitopes rather than linear epitopes. The findings are in accordance with Iorio’s that most of the selected monoclonal antibodies bind to HN, but only when it has been neither reduced nor boiled [47]. Which epitopes are recognized by our VHHs and do the epitopes different from previously reported need further investigation.

The NDV HN antibodies can be separated into two groups with respect to their mechanism of neutralization, namely, those that prevent receptor recognition and those that do not [46]. And evidence for a multiple-antibody mechanism of neutralization has been gathered in several systems with both polyclonal and monoclonal antibodies [48]. In the present study, as shown in Fig. 7, five of the seven VHHs displayed neutralizing activity against the virulent NDV F48E9, but the neutralizing titers were significantly lower than that of positive serum. Some syncytia could be observed on cells at 36–72 h post-infection (data not shown). Iorio reported that complete neutralization of NDV required monoclonal antibodies to different sites of HN glycoprotein [49]. So the neutralizing activity of a single VHH may be limited. Apart from the above reason, the following aspects might be also account for the relatively poor neutralizing activity: 1) Antigenic difference exist between LaSota strain and F48E9 strain (Table 2) lead to the low antibody binding activity. 2) The epitopes recognized by these VHH were not the crucial region for the blockage of virus. Therefore, we will attempt to identify the epitopes recognized by these selected VHHs in our future work. 3) The antibodies targeting fusion protein (F protein) are reportedly more effective in preventing virus infection than the antibodies targeting the HN protein [50–53]. So the screening of the VHH targeting F protein or other viral proteins is an alternative way. 4) As mentioned previously, we presume that the smaller size of the VHH could enhance its tissue penetration and make it bind some cryptic epitopes. However, on the contrary, the smaller size may be unable to fully occupy the targeting site or form a steric hindrance to prevent virus infection. The multimerization of the selected VHHs may be a mechanism to enhance their function.

## Conclusions

In this study, we constructed a VHH yeast two-hybrid library. After two rounds of screening, seven VHHs targeted to HN protein were selected and characterized for the first time. These selected VHHs can specifically interact with the HN protein and inhibit the HA activity of the NDV. Most VHHs also showed a neutralizing activity. These VHHs have great potential for application in NDV diagnosis, therapy, and pathogenesis. Moreover, the VHHs screened on the basis of the yeast two-hybrid may be an effective tool to examine the mechanism of ND pathogenesis and vaccination.

## Additional file

Additional file 1: Table S1. Humoral immune response after immunization. Sera from IIama was collected, two-fold diluted and tested by HI using LaSota as antigen. Figure S1 Amplification of VHH through a nested PCR. (A) First round PCR to separate VH from VHH. The upper 900 bp bands represent the VH-CH1-Hinge-CH2 of conventional Abs (lane 1–8). The lower 600 bp bands represent the VHH-Hinge-CH2 of HCAbs (lane 1–8). (B) VHH amplified through nested PCR using 600 bp fragment recovered from first round PCR as template (lane 1–4). M in A and B was the DL2000 DNA marker. C in A and B represent the negative control. Figure S2 PCR identification of inserted VHH. 47 clones were randomly picked to determine the library functional diversity by PCR using universal primers T7 and 3’AD (Table 1). Meanwhile, Sterile water was used as negative controls. 45 clones have amplified the 500 bp VHH fragments (lane 1–47), while negative templates control haven’t amplified any bands (lane C). M indicated the DL2000 DNA marker. Figure S3 Detection of library capacity and library titer. (A) 10^-3^ dilution plating of the transformed cells calculated a library capacity of 1.25 × 10^7^ independent clones. (B) 10^-5^ dilution plating of the cultured library indicated a library titer of 3.45 × 10^8^ cfu/mL. Figure S4 Deduced amino acid aligment of 10 random picked VHH. Deduced amino acid sequences were analyzed according to the Kabat numbering. Differences in the sequences are pinked, and the dash represent the missing sequences. Two hallmark Cys residues are labeled by the thick-line boxes. The four conservative hallmark residues of VHH in FR2 are labeled by the dotted line boxes. Figure S5 pGBKT7-HN bait plasmid construction. (A) PCR was carried out to amplify a truncate HN gene (without transmembrane region) from La Sota strain. M, 5000 DNA marker. 1, Truncate HN. C, Negative control. (B) A truncate HN was cloned into pGBKT7 through *BamH* I and *Sal* I. M, 5000 DNA marker. 1, Double restriction enzyme digestion of pGBKT7-HN. Figure S6 pHSIE-VHH plasmid construction. (A) 7 positive VHH fragment were amplified from recovered positive clones containing pGADT7-VHH by PCR. M, 5000 DNA marker. 1–7, VHH 1–7. C, Negative control. (B) Double restriction enzyme digestion of pHSIE-VHHs. M, 5000 DNA marker. 1–7, pHSIE-VHH 1–7. Figure S7 Western blot analysis of bait protein expression. 2 mL of Y2HGold(pGBKT7-HN) culture liquid was extracted using yeast protein extraction reagent (Takara). c-Myc tag monoclonal antibody (1:4000 dilution) was used as first antibody and HRP-labeled goat anti-mouse antibody (1:5000) was used as second antibody. The immunoreactive was visualized with cECL Plus Western blotting detection reagent (CWBIO). (DOC 1129 kb)

## Abbreviations

ND: Newcastle disease
NDV: Newcastle disease virus
VHH: Variable domain of heavy chain of the heavy-chain antibody
HA: Haemagglutinin activity
HN: Haemagglutinin-neuraminidase protein
HI: Haemagglutination inhibition
SUMO: Small ubiquitin-related modifier
Y2H: Yeast two-hybrid
SD/-Leu: Synthetically defined medium lacking leucine
DDO/X/A: Double dropout medium lacking tryptophan and leucine and supplemented with X-α-Gal and Aureobasidin A
QDO/X/A: Quadruple dropout medium lacking adenine, histidine, tryptophan and leucine and supplemented with X-α-Gal and Aureobasidin A
PBS: Phosphate-buffered saline
IPTG: Isopropyl-D-thiogalactopyranoside
TMB: Tetramethylbenzidine
VN: Virus neutralization assay
CDR: Complementary determining region
FR: Framework region
