# Supplementary material for: Construction of a camelid VHH yeast two-hybrid library and the selection of VHH against haemagglutinin-neuraminidase protein of the Newcastle disease virus
Source: BMC Vet Res. 2016 Feb 26;12:39. doi: 10.1186/s12917-016-0664-1 (PMC4769559; doi:10.1186/s12917-016-0664-1)
Supplement: Additional file 1: Table S1. — Humoral immune response after immunization. Sera from IIama was collected, two-fold diluted and tested by HI using LaSota as antigen. Figure S1 Amplification of VHH through a nested PCR. (A) First round PCR to separate VH from VHH. The upper 900 bp bands represent the VH-CH1-Hinge-CH2 of conventional Abs (lane 1–8). The lower 600 bp bands represent the VHH-Hinge-CH2 of HCAbs (lane 1–8). (B) VHH amplified through nested PCR using 600 bp fragment recovered from first round PCR as template (lane 1–4). M in A and B was the DL2000 DNA marker. C in A and B represent the negative control. Figure S2 PCR identification of inserted VHH. 47 clones were randomly picked to determine the library functional diversity by PCR using universal primers T7 and 3’AD (Table 1). Meanwhile, Sterile water was used as negative controls. 45 clones have amplified the 500 bp VHH fragments (lane 1–47), while negative templates control haven’t amplified any bands (lane C). M indicated the DL2000 DNA marker. Figure S3 Detection of library capacity and library titer. (A) 10-3 dilution plating of the transformed cells calculated a library capacity of 1.25 × 10^7^ independent clones. (B) 10-5 dilution plating of the cultured library indicated a library titer of 3.45 × 10^8^ cfu/mL. Figure S4 Deduced amino acid aligment of 10 random picked VHH. Deduced amino acid sequences were analyzed according to the Kabat numbering. Differences in the sequences are pinked, and the dash represent the missing sequences. Two hallmark Cys residues are labeled by the thick-line boxes. The four conservative hallmark residues of VHH in FR2 are labeled by the dotted line boxes. Figure S5 pGBKT7-HN bait plasmid construction. (A) PCR was carried out to amplify a truncate HN gene (without transmembrane region) from La Sota strain. M, 5000 DNA marker. 1, Truncate HN. C, Negative control. (B) A truncate HN was cloned into pGBKT7 through BamH I and Sal I. M, 5000 DNA marker. 1, Double restriction enzyme digestion of pGBKT7-HN. Figur [file 12917_2016_664_MOESM1_ESM.doc]

**Supplement Table. S1**

| Immunization times | 1 st | 2 nd | 3 rd | 4 th | 5 th |  |
| --- | --- | --- | --- | --- | --- | --- |
| Day post 1 st immunization (day) | 0 | 14 | 28 | 42 | 56 | 70 |
| HI titer (Log2) | ND^a^ | ND | 8 | ND | ND | 10 |

“a”, not detected

Supplement Table.S1 Humoral immune response after immunization. Sera from IIama was collected, two-fold diluted and tested by HI using LaSota as antigen.

**Supplement Fig. S1**


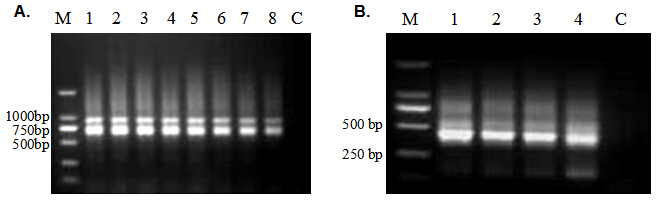


Supplement Fig.S1 Amplification of VHH through a nested PCR. (A) First round PCR to separate VH from VHH. The upper 900 bp bands represent the VH-CH1-Hinge-CH2 of conventional Abs (lane 1-8). The lower 600 bp bands represent the VHH-Hinge-CH2 of HCAbs (lane 1-8). (B) VHH amplified through nested PCR using 600 bp fragment recovered from first round PCR as template (lane 1-4). M in A and B was the DL2000 DNA marker. C in A and B represent the negative control.

**Supplement Fig. S2**


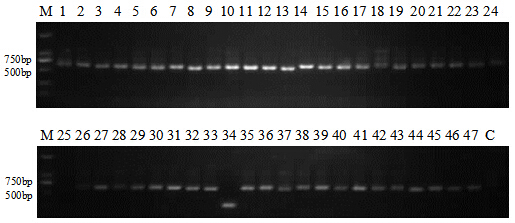


Supplement Fig.S2 PCR identification of inserted VHH. 47 clones were randomly picked to determine the library functional diversity by PCR using universal primers T7 and 3’AD (Table 1). Meanwhile, Sterile water was used as negative controls. 45 clones have amplified the 500 bp VHH fragments (lane 1-47), while negative templates control haven’t amplified any bands (lane C). M indicated the DL2000 DNA marker.

**Supplement Fig. S3**


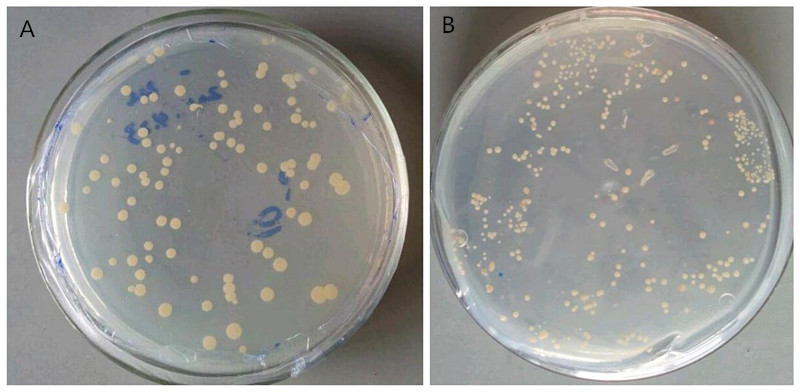


Supplementary Fig.S3 Detection of library capacity and library titer. (A) 10^-3^ dilution plating of the transformed cells calculated a library capacity of 1.25 × 10^7^ independent clones. (B) 10^-5^ dilution plating of the cultured library indicated a library titer of 3.45 × 10^8^ cfu/mL.

**Supplement Fig. S4**


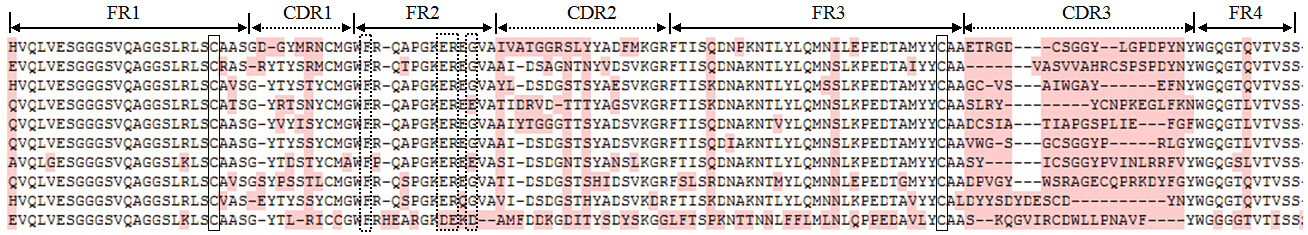


Supplement Fig.S4 Deduced amino acid aligment of 10 random picked VHH. Deduced amino acid sequences were analyzed according to the Kabat numbering. Differences in the sequences are pinked, and the dash represent the missing sequences. Two hallmark Cys residues are labeled by the thick-line boxes. The four conservative hallmark residues of VHH in FR2 are labeled by the dotted line boxes.

**Supplement Fig.S5**


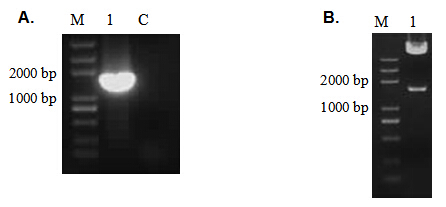


Supplement Fig.S5 pGBKT7-HN bait plasmid construction. (A) PCR was carried out to amplify a truncate HN gene (without transmembrane region) from La Sota strain. M, 5000 DNA marker. 1, Truncate HN. C, Negative control. (B) A truncate HN was cloned into pGBKT7 through *BamH* I and *Sal* I. M, 5000 DNA marker. 1, Double restriction enzyme digestion of pGBKT7-HN.

**Supplement Fig.S6**


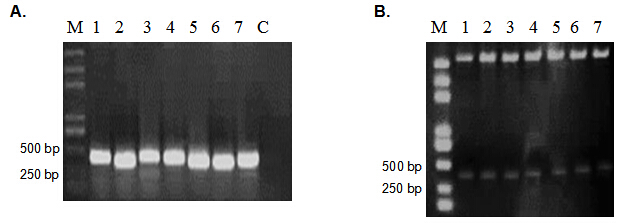


Supplement Fig.S6 pHSIE-VHH plasmid construction. (A) 7 positive VHH fragment were amplified from recovered positive clones containing pGADT7-VHH by PCR. M, 5000 DNA marker. 1-7, VHH1-7. C, Negative control. (B) Double restriction enzyme digestion of pHSIE-VHHs. M, 5000 DNA marker. 1-7, pHSIE-VHH 1-7.

**Supplement Fig.S7**


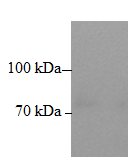


Supplement Fig.S7 Western blot analysis of bait protein expression. 2 mL of Y2HGold(pGBKT7-HN) culture liquid was extracted using yeast protein extraction reagent (Takara). c-Myc tag monoclonal antibody (1:4000 dilution) was used as first antibody and HRP-labeled goat anti-mouse antibody (1:5000) was used as second antibody. The immunoreactive was visualized with cECL Plus Western blotting detection reagent (CWBIO).
